# The role of surgical intervention for isolated breast cancer liver metastasis: Results of case‐control study with comparison to medical treatment

**DOI:** 10.1002/cam4.3117

**Published:** 2020-05-12

**Authors:** Jiahuai Wen, Feng Ye, Fengfeng Xie, Dan Liu, LeZhen Huang, Chen Fang, Shaowen Zhong, Liping Ren

**Affiliations:** ^1^ Department of Breast Oncology Guangdong Hospital of Traditional Chinese Medicine Guangzhou China; ^2^ The Standardized Training of Residents Sun Yat‐Sen University Cancer Center Guangzhou China; ^3^ Department of Breast Oncology Sun Yat‐Sen University Cancer Center State Key Laboratory of Oncology in South China Collaborative Innovation Center for Cancer Medicine Guangzhou China

**Keywords:** breast cancer, liver metastasis, surgical intervention

## Abstract

**Background:**

Combined with systemic therapy, the surgical intervention for breast cancer liver metastases (BCLM) is increasingly accepted but lacks convincing evidence. The aim of this study was to evaluate the disease control efficacy of hepatic surgery in isolated BCLM patients.

**Methods:**

Between 2012 and 2017, metastatic breast cancer patients with isolated liver metastasis and regular follow‐up were identified. Cohort design was conducted to compare the progression‐free survival (PFS) between the surgical and nonsurgical BCLM patients. Univariate analysis and multivariate Cox regression survival analyses were performed to identify significant prognostic factors.

**Result:**

In all, 148 isolated BCLM patients were enrolled and 95 participants received hepatic surgery for metastatic lesions. With median follow‐up of 36.47 months, there was no significant difference between hepatic surgical group and nonsurgical group for PFS (median PFS: 11.17 months vs 10.10 m, *P* = .092). Based on the multivariate analysis, the disease‐free interval (DFI) was an independent prognostic factor for isolated BCLM patients. Among the surgical group, BCLM patients who had ideal response after first salvage systemic treatment experienced the best long‐term survival (median PFS: 14.20 months).

**Conclusion:**

For isolated BCLM patients with ideal response in first‐line medical treatment, surgical intervention (hepatectomy, radiofrequency ablation) combining with systemic treatment could bring improved progression‐free survival compared to sole systemic treatment, indicating that hepatic surgery may be considered as a therapeutic choice for selected isolated BCLM patients in clinical practice.

## Graphical abstract

In highly selected patients, surgical intervention (hepatectomy, radiofrequency ablation) combining with systemic treatment could bring improved progression‐free survival compared to sole systemic treatment, indicating that hepatic surgery may be considered as a therapeutic choice for selected isolated BCLM patients in clinical practice.

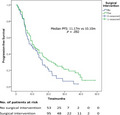

## 1 INTRODUCTION

Breast cancer is the most common cancer in female worldwide.^1^ In the United States, it is estimated that more than 270 000 new cases and 40 000 deaths occurred in 2019.^2^ Despite the improvement of breast cancer treatment, approximately 30% of patients would develop distant metastases during the course of disease.^3^ The third most commonly affected site is liver and 5%‐15% of patients have oligometastatic breast cancer liver metastases (BCLM).^4^, ^5^

According to the National Comprehensive Cancer Network (NCCN) and 4th ESO–ESMO International Consensus Guidelines for Advanced Breast Cancer (ABC4) for advanced breast cancer, the systemic therapies (such as cytotoxic chemotherapy and hormonal agents) are the recommended therapeutic strategy and could bring the improvement of survival for these patients.^6^, ^7^ However, merely 50% of metastatic lesions respond to the palliative therapy,^8^ and drug resistance is inevitable due to the biological feature of cancer cells. Thus, more concern is focused on the surgical intervention (hepatectomy, radiofrequency ablation) for isolated metastatic focus, but its survival benefit remains debatable.

Previous studies pointed out that hepatic surgeries for isolated liver metastasis were safe in advanced colorectal carcinoma patients.^9^, ^10^ Both hepatectomy and radiofrequency ablation could bring similar survival outcome among patients with solitary metastatic lesion,^11^, ^12^ and the 5‐year survival rates could achieve as high as 40%‐70%.^13^ However, the biological behavior of breast cancer differs from that of colorectal cancer,^14^ and little convincing evidence indicating survival advantage exists since only small sample‐sized, single‐cohort studies were available. Small retrospective studies have reported a median survival of 30‐70 months and 5‐year overall survival rates of 33% in BCLM treated with hepatectomy.^15^, ^16^, ^17^

The aim of this study was to evaluate the progression‐free survival of isolated BCLM patients treated with surgical intervention (hepatectomy, radiofrequency ablation) plus systemic therapy and to compare it with sole systemic therapy. Additionally, we attempt to identify the potential subgroup of BCLM patients benefited from the hepatic surgical intervention.

## 2 MATERIALS AND METHODS

### 2.1 Study design and population

The present study was retrospective cohort research incorporating consecutive advanced breast cancer patients from January 2012 to December 2017 at Sun Yat‐Sen University Cancer Center. Enrolled participants had to meet the following eligibility criteria: (1) pathologically and/or radiographically confirmed liver metastasis; (2) hepatic lesion ≤3 cm and number of lesion ≤ 2 in one hepatic segment;(3) female; (4) received first‐line salvage chemotherapy and/or endocrine therapy as initial treatment after the diagnosis of metastatic status; and (5) liver function classification of Child‐Pugh A or B. Exclusion criteria were as followed: (1) concomitant nonmammary malignancy; (2) extrahepatic metastasis before hepatic surgical intervention; (3) extrahepatic metastasis at enrollment; (4) adjacent organ involvement or cancer embolus in portal system; (5) no regular physical exam, laboratory examination, or imaging examination (mammogram, ultrasound) during the follow‐up; and (6) not enough data can be extracted.

The written informed consent for noninvasive retrospective study was obtained for each participant at the first hospital admission. The study protocol was approved by the institutional review board of Sun Yat‐Sen University Cancer Center before the initiation of the study.

### 2.2 Data collection and variables

Clinicopathologic data of each participant were screened and collected through electronic medical record (EMR) by two co‐authors, including study population characteristics (age, malignancy family history), breast cancer characteristics (pathological type, histologic grade, preliminary TNM stage, hormonal receptor status (estrogen receptor/ER, progesterone receptor/PR), human epidermal growth factor receptor‐2 (HER‐2) status, and Ki‐67 scores), hepatic metastases details (date, location, number, and size), first‐line salvage treatment regimens, and surgical intervention. The details of disease recurrence (date, recurrent location, and number) were also recorded. The therapeutic evaluation was assessed after the salvage systemic treatment based on the RECIST 1.1 criteria, respectively (Complete Response (CR): Disappearance of all target lesions, Partial Response (PR) ≥30% decrease in sum of all target lesions dimension, Progressive Disease (PD): new lesions or ≥20% increase in smallest sum of target lesions, and Stable Disease (SD): when sum of all target lesions does not qualify for CR/PR/PD).

### 2.3 Assessment and follow‐up

All the enrolled patients were regularly followed and received abdominal CT/MR scan every 2‐4 months for endocrine therapy or every two to four cycles for chemotherapy according to the ABC4 guideline. Progression‐free survival (PFS) was defined as the length of time between the diagnosis of hepatic metastasis and the disease progression in nonsurgical group or disease recurrence in surgical group.

### 2.4 Statistical analysis

Student t test or Mann‐Whitney test was performed for comparison of continuous variables, and categorical variables were compared using x^2^ or the Fisher exact test. The disease‐free interval (DFI) was defined as the interval from the primary treatment of a malignancy to the diagnosis of recurrence (contralateral primary breast cancer, locoregional or distant recurrence). DFIs were only calculated among non–de novo breast cancer patients who developed recurrent disease after adjuvant treatment. Patients who lost to follow‐up or did not suffer the event by the end of the study were defined as censored case at the time of last follow‐up. The primary outcome was PFS, which was estimated using the Kaplan‐Meier method and compared by the log‐rank test. The Cox proportional hazard regression model was utilized to identify potential factors associated with PFS and control confounding, calculating hazard ratios (HR) with corresponding 95% confidence intervals (95% CI). Subgroup analyses were conducted to evaluate the progression‐free survival benefit of hepatic surgery based on various conditions. SPSS 19.0 (IBM) was used for all analyses, and two‐tailed *P* ≤ .05 was considered as statistical significance.

## 3 RESULTS

### 3.1 Clinicopathologic characteristics of the study participants

A total of 327 isolated BCLM patients were identified according to the inclusion criteria, and 148 patients were finally enrolled in the study analyses (Supplement Figure S1). Baseline clinicopathologic characteristics were shown in the Table 1. Nineteen patients were de novo stage IV breast cancer patients, and the remaining participants (n = 129) suffered isolated hepatic metastases during the postoperative follow‐up. Among the preliminary nonadvanced participants, the majority were classified as either T1 or T2 (n = 121, 93.7%) and 6.3% participants were T3 or T4 staging. Regional lymph node metastases occurred in majority of patients (n = 96, 74.6%). The proportion of EP/PR positive and HER2 positive were 66.9% and 43.9% in the overall cohort, respectively, and 43.9% of participants were ER/PR+, HER2‐ subtype and triple‐negative subtype (ER/PR‐, HER2‐) only accounted for 12.2%. Majority of patients (63.5%) received chemotherapy in the first‐line salvage treatment, and almost all HER2‐positive BCLM patients (95%) received anti‐HER2 agents treatment.

**TABLE 1:** Characteristics of isolated BCLM patients stratified by treatment group

	Overall cohort (n = 148)	Surgical cohort (n = 95)	Nonsurgical group (n = 53)	*P* value
Age at BC diagnosis	44.94 ± 8.72	45.37 ± 9.01	44.13 ± 8.19	.410
Age				
≤35	21 (14.2%)	14 (14.7%)	7 (13.2%)	.798
>35	127 (85.8%)	81 (85.3%)	46 (86.8%)	
Age at BCLM	47.60 ± 9.35	48.12 ± 9.81	46.66 ± 8.47	.639
T staging^a^				.609
T1	36 (27.9%)	21 (25.3%)	15 (32.7%)	
T2	85 (65.8%)	56 (67.5%)	29 (63.0%)	
T3	6 (4.7%)	4 (4.8%)	2 (4.3%)	
T4	2 (1.6%)	2 (2.4%)	0 (0%)	
N staging^a^				.766
N0	33 (25.4%)	23 (27.7%)	10 (21.7%)	
N1	55 (42.3%)	33 (39.8%)	22 (47.8%)	
N2	20 (15.4%)	14 (16.9%)	6 (13.0%)	
N3	21 (16.9%)	13 (15.6%)	8 (17.4%)	
Preliminary stage				.993
1	17 (11.5%)	11 (11.6%)	6 (11.3%)	
2	68 (45.9%)	43 (45.3%)	25 (47.2%)	
3	44 (29.7%)	29 (30.5%)	15 (28.3%)	
4	19 (12.9%)	12 (12.6%)	7 (13.2%)	
ER/PR status				.196
Negative	49 (33.1%)	35 (36.8%)	14 (26.4%)	
Positive	99 (66.9%)	60 (63.2%)	39 (73.6%)	
HER‐2 status				.803
Negative	83 (56.1%)	54 (56.8%)	29 (54.7%)	
Positive	65 (43.9%)	41 (43.2%)	24 (45.3%)	
Intrinsic Subtype				.524
ER/PR+,HER2‐	65 (43.9%)	40 (42.1%)	25 (47.2%)	
ER/PR+,HER2+	34 (23.0%)	20 (21.1%)	14 (26.4%)	
ER/PR‐,HER2+	31 (20.9%)	21 (22.1%)	10 (18.9%)	
ER/PR‐,HER2‐	18 (12.2%)	14 (14.7%)	4 (7.5%)	
DFI for non–de novo advanced patients^a^	36.47 ± 32.24	37.78 ± 34.05	34.18 ± 28.98	
≤24 months	57 (47.8%)	35 (42.2%)	22 (47.8%)	.608
>24 months	72 (52.2%)	48 (57.8%)	24 (52.2%)	
1st‐line salvage treatment				.381
Endocrine therapy	54 (36.5%)	36 (37.9%)	18 (34.0%)	
Chemotherapy	94 (63.5%)	59 (62.1%)	35 (66.0%)	
Anti‐HER2 agent^b^	63 (95.4%)	40 (95.1%)	23 (95.8%)	
Mean PFS/months	12.90 ± 10.63	13.60 ± 11.62	11.64 ± 8.54	.224

Abbreviations: BC, breast cancer; BCLM, breast cancer liver metastasis; DFI, disease‐free interval; ER, estrogen receptor; HER‐2, human epidermal growth factor receptor‐2; PFS, progress‐free survival; PR, progesterone receptor.

^a^ For the de novo advanced breast cancer patients (n = 19), the T staging, N staging, and DFI information was not collected.

^b^ Only among BCLM patients with HER2‐positive.

Among the enrolled participants, 64.1% (n = 95) of BCLM patients underwent hepatic surgical intervention (hepatectomy: n = 12, radiofrequency ablation: n = 83) plus postoperative systemic therapy and were classified as the surgical group, the rest (n = 53) only received the systemic therapy (nonsurgical group). No significant difference of clinicopathologic characteristic was observed between surgical group and nonsurgical group. Among the surgical group, there were 56 patients who received first‐line salvage chemotherapy and/or endocrine therapy before the hepatic surgical intervention with median duration of 14.60 months, and 41 patients obtained ideal response (CR: n = 2, PR: n = 33, SD: n = 6), while the remaining patients (n = 15) progressed under the first‐line salvage treatment before hepatic surgical intervention.

### 3.2 Long‐term clinical outcome

**FIGURE 1:**
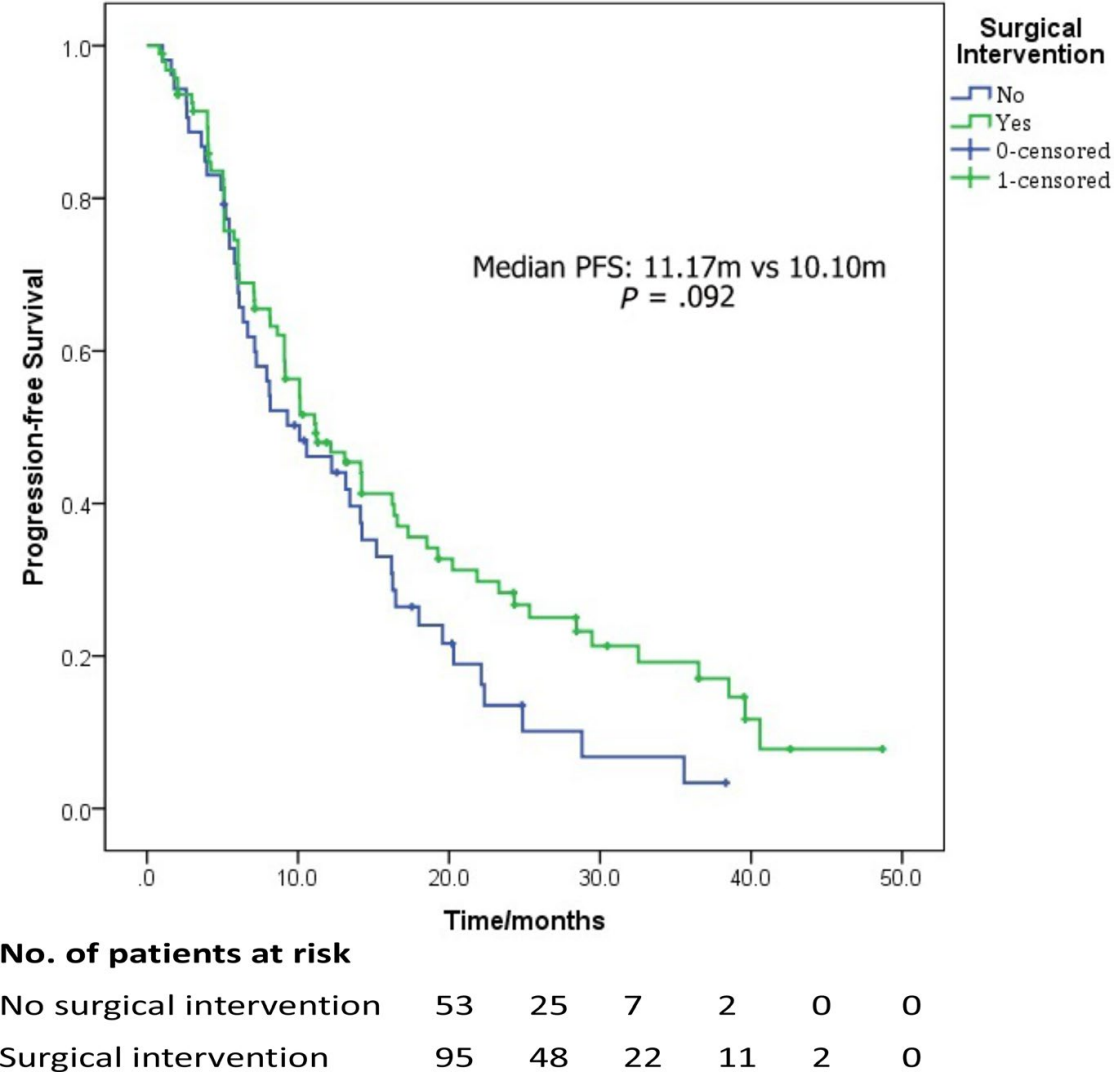
Progression‐free survival comparison between systemic treatment plus surgical intervention (surgical group) and sole systemic treatment (nonsurgical group) for patients with isolated breast cancer liver metastasis

The mean follow‐up time was 36.47months and the median PFS for the entire cohort was 10.57 (95% CI: 7.48‐13.66) months. In the total of 95 BCLM patients receiving hepatic surgical intervention, there were 69 patients suffered recurrent events at the end of the follow‐up (hepatic recurrence in 62 patients and extrahepatic recurrence in 7 patients), and the median PFS was 11.17 (95% CI: 6.91‐15.42) months. Survival curve indicated the tendency of PFS benefit in the surgical group, but no statistical difference of the median PFS was confirmed between surgical group and nonsurgical group (HR: 0.72, 95% CI: 0.49‐1.06, *P* = .09, Figure 1). Supplement Table S1 compared the clinical characteristics between isolated BCLM patients with or without disease progression in the surgical group, and the result indicated that BCLM patients receiving hepatectomy or having ideal response in the systemic treatment (non‐DP) experienced lower risk of disease progression, but statistical significance was not proven (*P* = .06 and .09).

### 3.3 Identification of independent parameters

**FIGURE 2:**
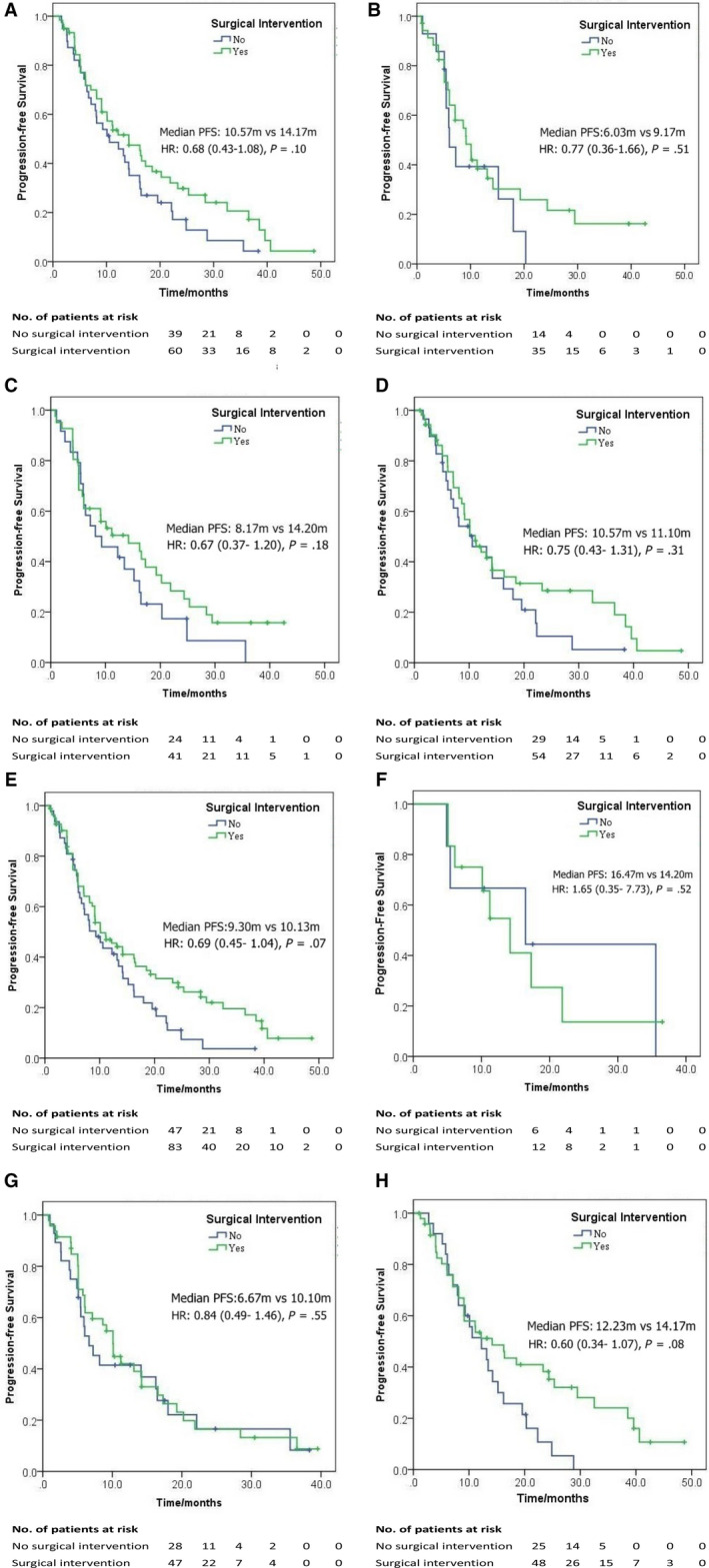
Subgroup analyses of progression‐free survival for isolated breast cancer liver metastasis patients. A, ER/PR‐positive, (B) ER/PR‐negative, (C)HER2‐positive, (D)HER2‐negative, (E) Preliminary stage 1‐3, (F) Preliminary stage 4, (G) DFI ≤24 mo, and (H) DFI >24 mo

In the univariate and COX multivariate analyses evaluating the prognostic factors of BCLM patients (Table 2), the results indicated that previous longer DFI was the only parameter associating with better PFS (HR: 0.66, 95% CI: 0.44‐0.99, *P* = .05). Nevertheless, age, preliminary stage, ER/PR status, HER‐2 status, and hepatic surgery did not significantly influence the long‐term outcome of isolated BCLM patients (Table 2, all *P* > .05). In the subgroup survival analyses based on the covariates, the result showed that hepatic surgery could not significantly prolong the PFS in various situation (Figure 2A‐H). However, better PFS tendency was observed in ER/PR‐positive and HER2‐positive BCLM patients (Figure 2A,C), while no similar tendency was observed in ER/PR‐negative or HER2‐negative subgroup (Figure 2B,D). When considering the intrinsic subtypes of breast cancer according to the immunohistochemical status, superior PFS was found in the surgical group of luminal subtype (ER/PR positive, HER2 negative), but no statistical significance was proven (HR: 0.63, 95% CI: 0.86‐2.92, *P* = .13, Supplement Figure S2).

**TABLE 2:** Univariate and multivariate analyses of PFS with Cox proportional hazards in isolated BCLM patients (N = 148)

Variable	Univariate analysis		Multivariate analysis	
	HR (95% CI)	*P* value	HR (95% CI)	*P* value
Age at BC diagnosis (≤35 vs >35)^a^	1.02 (0.99‐1.04)	.15	1.13 (0.65‐ 1.98)	.67
Preliminary stage (Non–IV stage vs IV stage)^a^	1.08 (0.88‐1.34)	.46	1.59 (0.83‐ 3.06)	.12
ER/PR status (Positive vs Negative)^a^	1.18 (0.79‐1.75)	.42	1.16 (0.76‐1.81)	.46
HER‐2 status (Positive vs Negative)^a^	0.98 (0.67‐1.41)	.88	1.04 (0.69‐1.56)	.85
DFI (>24 mo vs ≤24 mo)^a^	0.74 (0.59‐0.97)	.04	0.66 (0.43‐0.99)	.05
Hepatic surgery (Yes vs No)^a^	0.73 (0.49‐1.06)	.09	0.72 (0.49‐1.05)	.09

Abbreviations: BC, breast cancer; BCLM, breast cancer liver metastasis; CI, confidence interval; DFI, disease‐free interval; ER, estrogen receptor; HER‐2, human epidermal growth factor receptor‐2; HR, hazard ratio; PFS, progress‐free survival; PR, progesterone receptor.

^a^ The last level of each variable was chosen as the reference category for all regression analyses.

Furthermore, the univariate and COX multivariate analyses were performed to identify the potential factors associating the therapeutic effect among the surgical cohort (Table 3). The result indicated that the DFI and the therapeutic evaluation of preoperative systemic treatment were the independent prognostic factors for progression‐free survival of the above patients (HR: 0.38 (95% CI: 0.16‐0.88) and 0.23 (95% CI: 0.10‐0.51), respectively, both *P* < .05).

**TABLE 3:** Univariate and multivariate analyses of PFS in isolated BCLM patients receiving hepatic surgery (N = 95)

Variable	Univariate analysis		Multivariate analysis	
	HR (95% CI)	*P* value	HR (95% CI)	*P* value
Age at BC diagnosis (≤35 vs >35)^a^	1.02 (0.99‐1.05)	.19	1.02 (0.98‐1.06)	.35
Preliminary stage (Non–IV stage vs IV stage)	0.75 (0.42‐1.86)	.75	0.66 (0.25‐1.75)	.40
ER/PR status (Positive vs Negative)^a^	1.17 (0.71‐1.91)	.54	1.90 (0.89‐4.06)	.10
HER‐2 status (Positive vs Negative)^a^	1.00 (0.62‐1.60)	.98	1.19 (0.57‐2.45)	.64
DFI (>24 mo vs ≤24 mo)	0.67 (0.41‐1.09)	.11	0.38 (0.16‐0.88)	.002
Hepatic intervention (hepatectomy vs radiofrequency ablation)	2.01 (0.87‐4.66)	.10	1.50 (0.39‐5.84)	.56
Preoperative systemic treatment	1.02 (0.63‐1.64)	.95	1.03 (0.60‐1.76)	.92
Preoperative evaluation (Non‐DP vs DP)	0.34 (0.17‐0.69)	.003	0.23 (0.10‐0.51)	.001

Abbreviations: BC, breast cancer; BCLM, breast cancer liver metastasis; CI, confidence interval; DFI, disease‐free interval; DP, disease progression; ER, estrogen receptor; HER‐2, human epidermal growth factor receptor‐2; HR, hazard ratio; PFS, progress‐free survival; PR, progesterone receptor.

^a^ The last level of each variable was chose as the reference category for all regression analyses.

**FIGURE 3:**
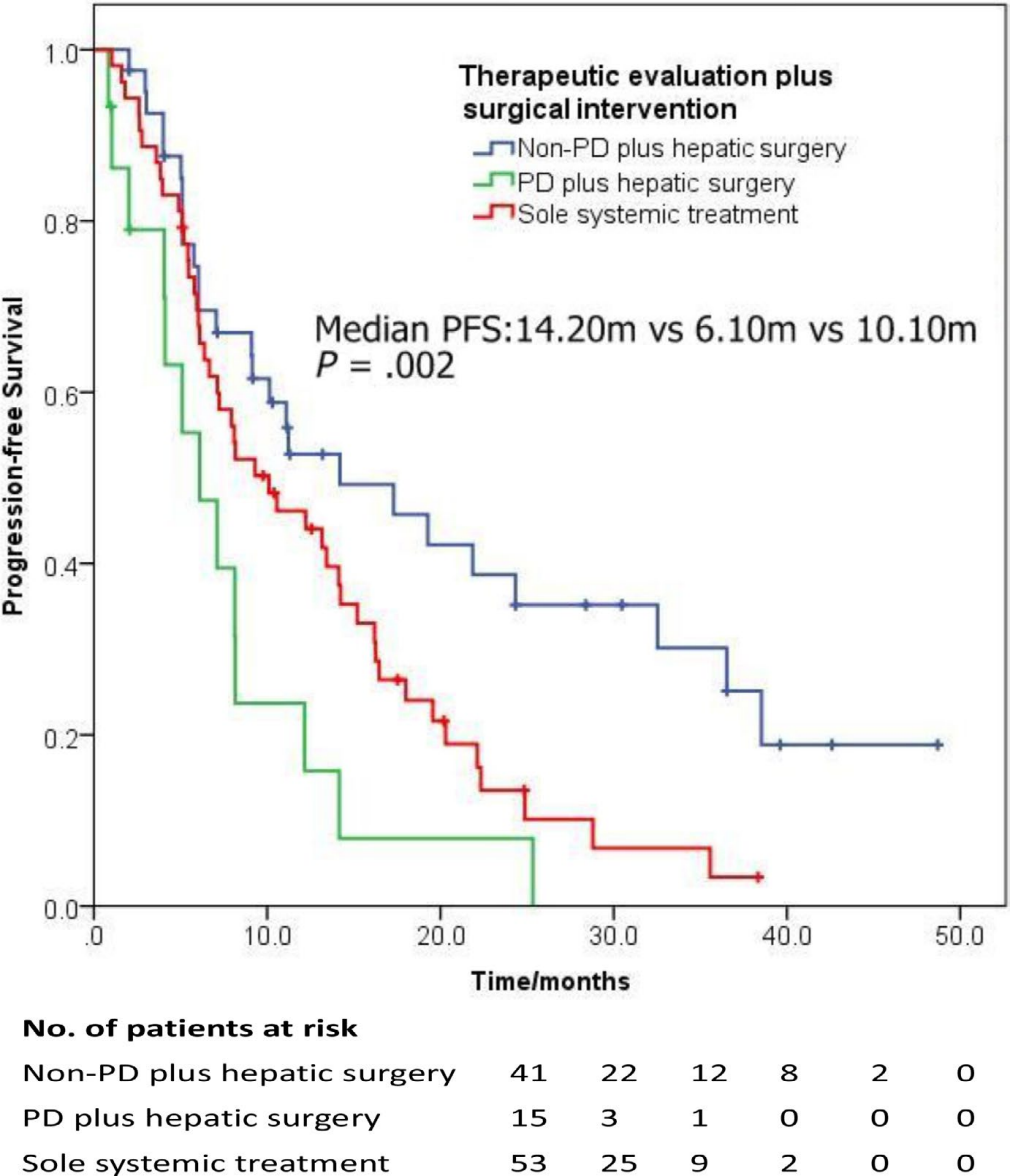
The combined effect of hepatic surgical intervention and preoperative therapeutic evaluation among isolated BCLM patients

**FIGURE 4:**
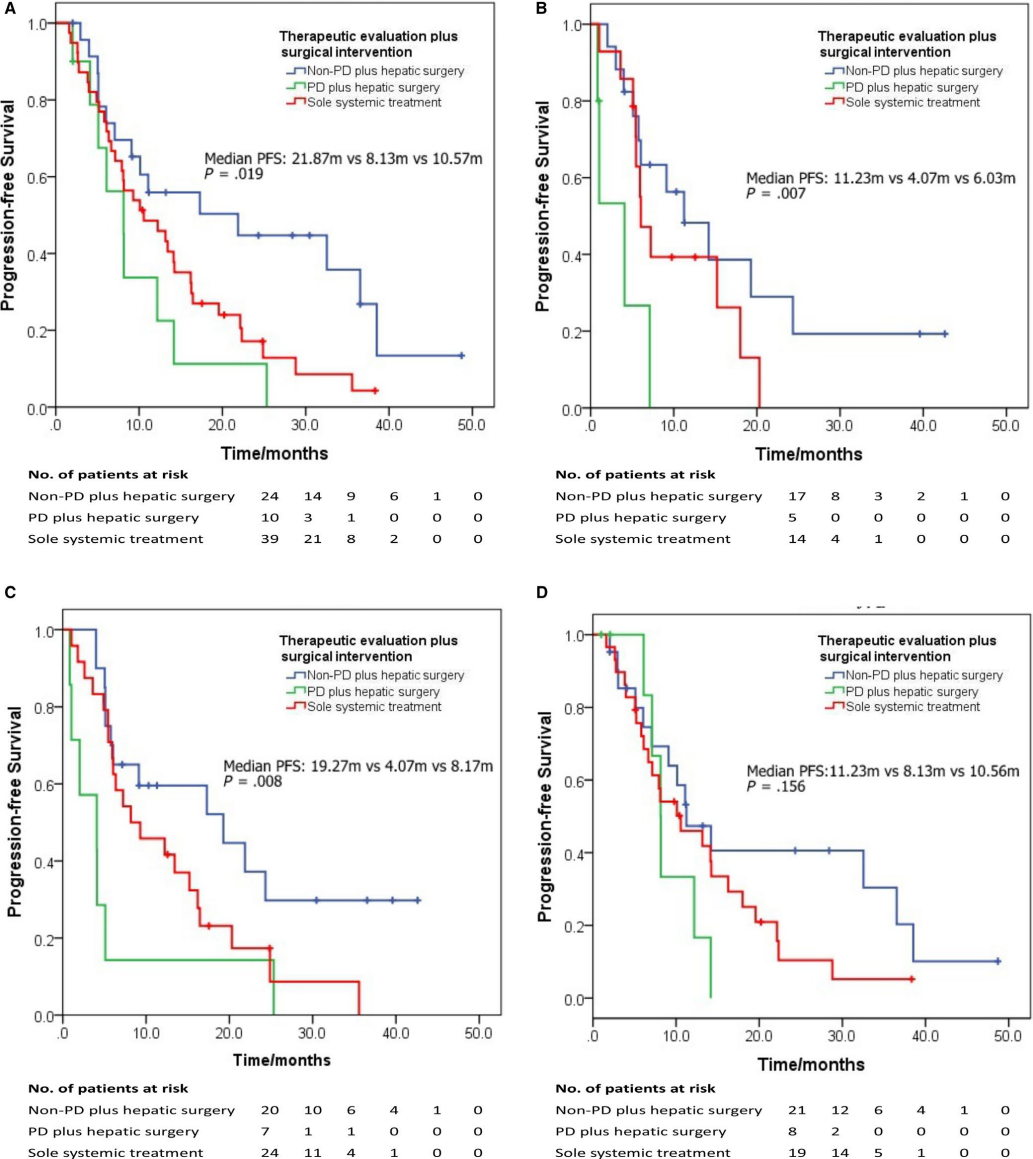
The subgroup analyses of progression‐free survival for isolated breast cancer liver metastasis patients. A, ER/PR‐positive, (B) ER/PR‐negative, (C) HER2‐positive, and (D) HER2‐negative

In order to assess the combined effect of hepatic surgical intervention and preoperative therapeutic evaluation, we further subdivided the enrolled participants into three groups (Non‐DP plus surgical intervention, DP plus surgical intervention, and sole systemic therapy). The survival analysis found that patients with non‐DP evaluation and receiving hepatic surgery experienced best PFS, while the worse PFS was observed among the BCLM patients with DP evaluation (Figure 3, median PFS: 14.20 m vs 6.10 m vs 10.10 m, HR: 0.53, 95% CI: 0.37‐ 0.75, *P* = .001). The result indicated the potential therapeutic value of surgical intervention for isolated hepatic metastatic lesion when the systemic treatment worked. Furthermore, this PFS benefit was not influenced by ER/PR and HER2 status (Figure 4). In the subgroup analyses, isolated breast cancer patients with non‐DP experienced superior PFS than that with DP in the surgical group in ER/PR‐positive and ER/PR‐negative subgroups (HR: 0.39 and 0.57, both *P* < .05). Similarly, in the HER2‐positive and HER2‐negative subgroups, hepatic surgery could also bring improvement of PFS for patients with ideal disease control from medical treatment (HR: 0.44 and 0.61, both *P* < .05).

## 4 DISCUSSION

Despite the improved prognosis of breast cancer due to the development of surgical intervention and systemic therapy, approximately 12%‐15% of breast cancer patients would finally suffer isolated hepatic metastasis at the first disease recurrence,^18^ with median survival of merely 3‐15 months when no treatment provided.^19^ Metastatic cancer is considered as a systemic disease, and cytotoxic chemotherapy and endocrine therapy are the preferred treatment for BCLM in current guidelines. The aim of present study is to evaluate the therapeutic value of surgical intervention and identify prognostic parameters for BCLM patients.

The therapeutic benefit of surgical resection of isolated hepatic metastasis was raised in colorectal cancer patients,^20^, ^21^ and there is growing interest about employing surgical intervention for BCLM patients. Breast cancer patients with oligometastatic hepatic status and no extrahepatic disease may be potential candidates for metastasectomy.^22^ But disease of colorectal cancer liver metastases is relatively confined to the abdomen because the metastases spread to the liver via portal venous system and/or abdominal lymphatic channels, and hepatectomy could offer ideal control of disease. However, breast malignant cells spread to the liver via systemic hematogenous dissemination, and the benefit of surgical intervention in these patients is under debate.^12^, ^23^, ^24^ Previous meta‐analysis has indicated that radiofrequency ablation and hepatectomy for BCLM have similar therapeutic efficacy.^25^

In the present study, enrolled patients suffered relatively low tumor burden (solitary, hepatic lesion ≤3 cm, and number of lesions ≤2 in one hepatic segment), who may be potential candidate for hepatectomy/radiofrequency ablation. But the median PFS of BCLM patients receiving hepatic surgery was similar to that of patients only receiving systemic therapy (11.17 m vs 10.10 m), indicating that hepatic surgical intervention could not provide progression‐free survival benefit for overall BCLM patients. The clinicopathological features of enrolled patients were balanced and did not influence the decision of application of hepatectomy/radiofrequency ablation, thus the clinical decision was done due to clinical discretion and patient preference.

Notably, BCLM patients who gained clinical benefit from the first‐line salvage therapy experienced better PFS than those with disease progression evaluation plus hepatic surgery and the patients only received systemic treatment. Analogously, response to chemotherapy could predict the survival of colorectal liver metastasis patients after hepatectomy, and patients progressing during the preoperative chemotherapy experience 5‐year survival rate of only 8%.^26^ However, one retrospective study showed that BCLM patients with stable disease during preoperative systemic treatment experienced lower 5‐year survival rate than those who achieved ideal response.^15^ Breast cancer metastases are not confined to the portal venous drainage system and subclinical metastases might exist in extrahepatic tissue. Good clinical response of first‐line systemic treatment generally means ideal disease control systematically. Hepatectomy/radiofrequency ablation could bring better outcome because of the surgical removal of remnant malignant cells that might occur therapeutic resistance and source for relapse. Combined with our finding, we surmised that hepatic surgery after the systemic treatment could eliminate local tumor burden and provide further control of metastasis. This may help clinicians to reconsider the therapeutic strategy and discuss the value of surgery with patients in the clinical practice. If isolated BCLM patients could achieve relatively good response to the systemic treatment, those patients may benefit from the hepatic surgery and get better progression‐free survival.

Metastasectomy for BCLM was theoretically valuable due to the following reasons. Firstly, the oligometastasis represent a state of limited metastatic spread and local treatment may achieve potential cure.^27^ Without additional external stimuli, tumor cells might remain in a state of dormancy and do not develop into overt metastases in the secondary organ. Secondly, surgical removing chemotherapy‐resistant clones may improve the efficacy of systemic therapy.^28^ Some have advocated that BCLM could follow a similar management guideline as for colorectal liver metastases,^29^ but surgeons are historically reluctant to perform hepatic surgery for non colorectal/nonneuroendocrine liver metastasis patients. Previous multi‐institutional study found that 460 isolated BCLM patients receiving hepatectomy experienced 45 months median overall survival and 5‐years OS of 41%, approximating those obtain among colorectal liver metastases. Systematic review consisting 19 studies and 553 patients indicated that hepatectomy of isolated BCLM brings median OS as 40 months and the median 5‐year survival rate as 40%.^30^

Another parameter representing tumor biology is the disease‐free interval from the time of primary tumor treatment to the occurrence of hepatic metastases. Previous analyses have verified that better progression‐free survival associates with surgical treatment.^17^, ^24^ In our study, we confirmed that disease‐free interval was significantly associated with the PFS in the isolated BCLM patients receiving surgical intervention. The probable reason may be that longer disease‐free interval indicates low aggressive tumor biology, and surgical removal of metastatic lesion could improve the disease control beyond the medical treatment. However, other studies failed to demonstrate the association between DFI and survival benefit after hepatic surgery.^15^, ^16^ Moreover, due to the small sample size and the retrospective shortcoming, we tend to regard long‐DFI as a prognostic factor of hepatic surgery for isolated BCLM patients, not a decisive factor for treatment appliance.

Several limitations existed in our study. Firstly, as a single‐institution retrospective study, selection bias was inherent limitation. Secondly, the overall sample size was relatively small and could not completely reflect the causal inference between hepatic surgery and prognosis among isolated BCLM patients. It also restricted the statistical power of subgroup analyses. Furthermore, BCLM patients with missing data were excluded in the study, and part of these patients may experience severe illness, which could bias in the cohort being analyzed. Finally, systemic treatment for advanced disease changed during the long spanned time of patient enrollment, which might affect the overall therapeutic effect. In the future, randomized trials are needed to further verify the long‐term survival benefit of surgical intervention on isolated BCLM patients.

## 5 CONCLUSION

The present study indicated that addition of hepatic surgery (hepatectomy or radial ablation) could not bring significant improvement of PFS in isolated BCLM patients. However, specific patients who achieved ideal response in preoperative systemic therapy experienced PFS benefit from the hepatic surgery. Therefore, hepatic surgery may be incorporated into therapeutic recommendation for particular subgroup of isolated BCLM patients when more convincing evidence is available in the near future.

## CONFLICT OF INTEREST

The authors declare that they have no competing interests.

## AUTHORS' CONTRIBUTIONS

LP initiated the project. JW, FY, LZ generated and analyzed the data. FX was responsible for statistical analyzes. LZ, YC wrote the manuscript. JW, FY, ZW advised and corrected the manuscript. All authors read and approved the final manuscript.

## ETHICS APPROVAL AND CONSENT TO PARTICIPATE

All participants were provided written informed consent. Study was approved by Sun Yat‐Sen University Cancer Center.

## Supporting information

Table S1

Figure S1

Figure S2

## Data Availability

The datasets analyzed during the current study are available from the corresponding author on reasonable request.
