# Supplementary material for: The role of surgical intervention for isolated breast cancer liver metastasis: Results of case‐control study with comparison to medical treatment
Source: Cancer Med. 2020 May 12;9(13):4656–66. doi: 10.1002/cam4.3117 (PMC7333858; doi:10.1002/cam4.3117)
Supplement: Supplementary file 1 — Table S1 [file CAM4-9-4656-s001.docx]

Supplement Table 1. Clinicopathological features of isolated BCLM patients experiencing different prognosis among surgical group.

|  | No recurrence  (n= 26) | Recurrence  (n=69) | P value |
| --- | --- | --- | --- |
| Age at BC diagnosis | 44.31 ± 8.93 | 45.77 ± 9.08 | 0.48 |
| ≤ 35 | 4 (28.6%) | 10 (71.4%) | 0.91 |
| ＞ 35 | 22 (27.2%) | 59 (72.8%) |  |
| Age at BCLM | 47.37±10.21 | 48.40 ± 9.71 | 0.65 |
| T staging* |  |  | 0.38 |
| T1 | 7 (33.3%) | 14 (66.7%) |  |
| T2 | 15 (26.8%) | 41 (73.2%) |  |
| T3 | 0 (0%) | 4 (100.0%) |  |
| T4 | 0 (0%) | 2 (100.0%) |  |
| N staging* |  |  | 0.98 |
| N0 | 5 (21.7%) | 18 (78.3%) |  |
| N1 | 10 (30.3%) | 23 (69.7%) |  |
| N2 | 4 (28.6%) | 10 (71.4%) |  |
| N3 | 3 (23.1%) | 10 (76.9%) |  |
| Preliminary stage |  |  | 0.51 |
| 1 | 3 (27.3%) | 8 (72.7%) |  |
| 2 | 12 (27.9%) | 31 (72.1%) |  |
| 3 | 7 (24.1%) | 22 (75.9%) |  |
| 4 | 4 (33.3%) | 8 (66.7%) |  |
| ER/PR status |  |  | 0.84 |
| Negative | 10 (28.6%) | 25 (71.4%) |  |
| Positive | 16 (26.7%) | 44 (73.3%) |  |
| HER-2 status |  |  | 0.57 |
| Negative | 16 (29.6%) | 38 (70.4%) |  |
| Positive | 10 (24.4%) | 31 (75.6%) |  |
| DFI for non-IV stage patients* | 43.50±37.62 | 35.71±32.76 | 0.36 |
| ≤ 24 months | 8 (22.9%) | 27 (77.1%) | 0.52 |
| ＞ 24 months | 14 (39.2%) | 34 (70.8%) |  |
| Surgical intervention |  |  |  |
| Hepatectomy | 6 (50.0%） | 6 (50.0%) | 0.06 |
| Radiofrequency ablation | 20 (24.1%) | 63 (75.9%) |  |
| Preoperative systemic treatment |  |  |  |
| Yes | 17 (30.4%) | 39 (69.6%) | 0.43 |
| No | 9 (23.1%) | 30 (76.9%) |  |
| Preoperative evaluation ^#^ |  |  |  |
| Non-DP | 15 (36.6%) | 26 (63.4%) | 0.09 |
| DP | 2 (13.3%) | 13 (86.7%) |  |

*For the de novo advanced breast cancer patients, the T staging and N staging information were not collected.

^#^ Preoperative evaluation was performed only to those BCLM patients received 1^st^ line salvage chemotherapy and/or endocrine therapy before the hepatic surgical treatment
