# Supplementary figures and images for: The role of surgical intervention for isolated breast cancer liver metastasis: Results of case‐control study with comparison to medical treatment
Source: Cancer Med. 2020 May 12;9(13):4656–66. doi: 10.1002/cam4.3117 (PMC7333858; doi:10.1002/cam4.3117)

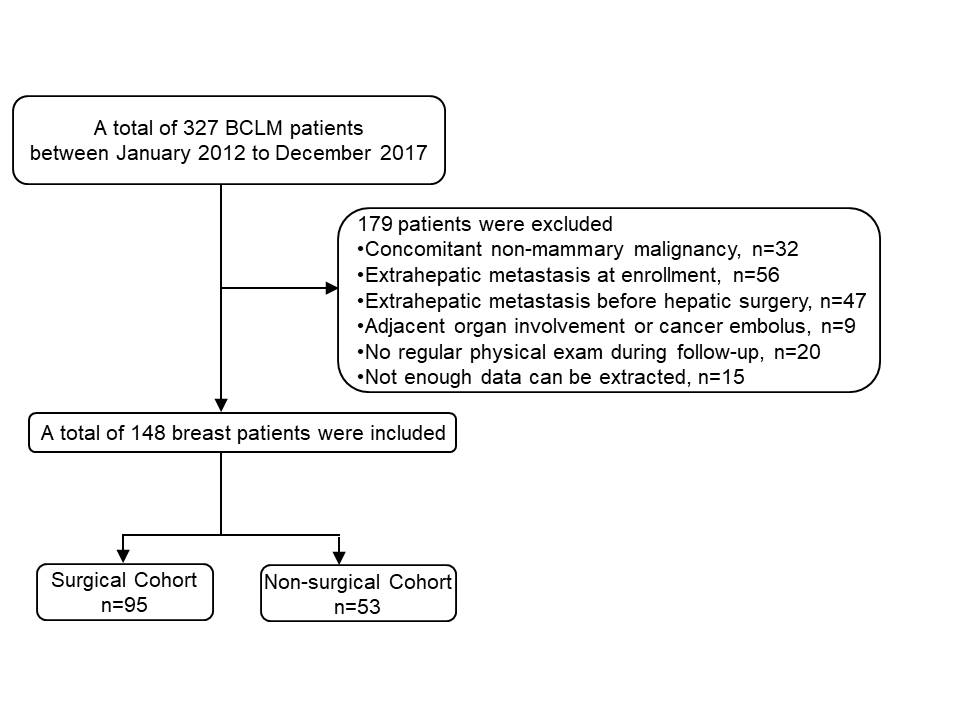

Supplement: Supplementary file 2 — Figure S1 [file CAM4-9-4656-s002.jpg]

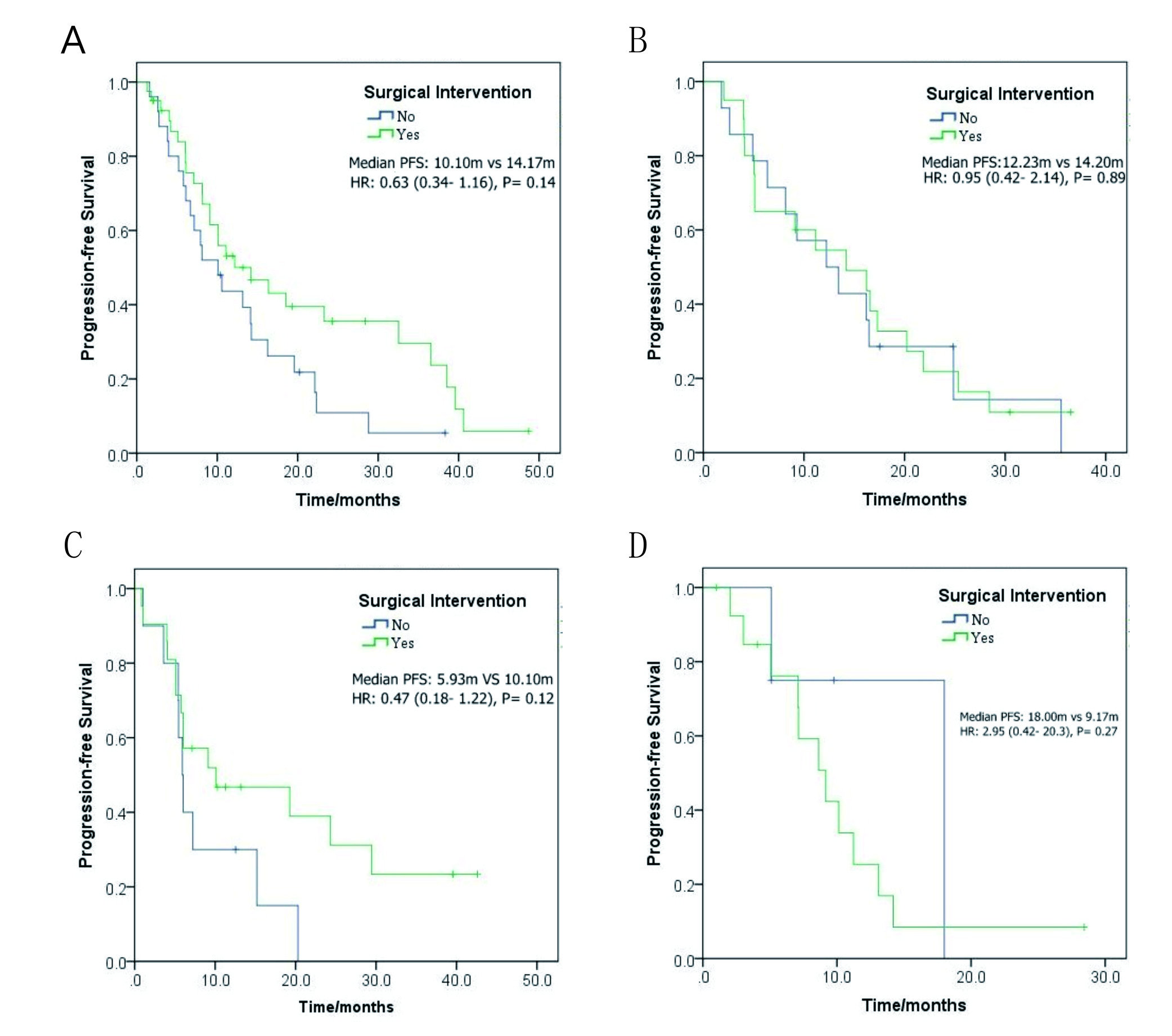

Supplement: Supplementary file 3 — Figure S2 [file CAM4-9-4656-s003.jpg]
